# Selected vs. Non-Selected Under-20 National Futsal Players: Differences between Physical Performance and Training Intensity Experienced in Training Camps

**DOI:** 10.3390/biology11030434

**Published:** 2022-03-11

**Authors:** Ana Filipa Silva, Francisco Tomás González-Fernández, Rafael Oliveira, Filipe Manuel Clemente, Pedro Bezerra, Chin-Hwai Hung, Yi-Wen Chiu, Cheng-Deng Kuo, Yung-Sheng Chen

**Affiliations:** 1 Escola Superior de Desporto e Lazer, Instituto Politécnico de Viana do Castelo, Rua Escola Industrial e Comercial de Nun’Álvares, 4900-347 Viana do Castelo, Portugal; anafilsilva@gmail.com (A.F.S.); filipe.clemente5@gmail.com (F.M.C.); pbezerra@esdl.ipvc.pt (P.B.); 2 Research Center in Sports Performance, Recreation, Innovation and Technology (SPRINT), 4960-320 Melgaço, Portugal; 3 The Research Centre in Sports Sciences, Health Sciences and Human Development (CIDESD), 5001-801 Vila Real, Portugal; rafaeloliveira@esdrm.ipsantarem.pt; 4 Department of Physical Education and Sport, Faculty of Education and Sport Sciences, Campus of Melilla, University of Granada, 52006 Melilla, Spain; francis.gonzalez.fernandez@gmail.com; 5 Sports Science School of Rio Maior-Polytechnic Institute of Santarém, 2040-413 Rio Maior, Portugal; 6 Life Quality Research Centre, 2040-413 Rio Maior, Portugal; 7 Instituto de Telecomunicações, Delegação da Covilhã, 1049-001 Lisboa, Portugal; 8 Department of Physical Education, Fu Jen Catholic University, New Taipei City 242, Taiwan; 088750@mail.fju.edu.tw (C.-H.H.); 055332@mail.fju.edu.tw (Y.-W.C.); 9 Department of Medical Research, Taipei Veterans General Hospital, Taipei 112, Taiwan; 10 Tanyu Research Laboratory, Taipei 112, Taiwan; 11 Department of Exercise and Health Sciences, University of Taipei, Taipei 111, Taiwan; 12 Exercise and Health Promotion Association, New Taipei City 241, Taiwan

**Keywords:** indoor football, sports, fitness, athletic performance, physical demands

## Abstract

**Simple Summary:**

In sports training, the physical profiles and performances between selected and non-selected players are not identical. The players’ specific characteristics, such as exercise performance, locomotion profiles, and physical exertions during training sessions, can be used to identify the successful achievement in sports. However, such information has not been revealed in futsal. This study followed a prospective cohort design between 2018 and 2019. In our study, under-20 male Taiwan national futsal players were assessed for their physical fitness in two separate time periods. The perceived responses of physical exertion, exercise heart rate responses, and locomotion profiles were recorded during each training camp. Quantitative data during eighteen training camps/competition periods were collected for comparison. Our findings demonstrated that the selected players had a superior performance in 15 m Sprint and 30 m Sprint, exercise HR responses, and locomotor demands compared to the non-selected players. Coaches and their staff could consider the information of the present study for identifying selected players.

**Abstract:**

The aim of this study was two-fold: (i) analyze the variations in the physical fitness of selected and non-selected under-20 male national futsal players; and (ii) analyze the variations in training intensity monitored during training camps. Thirty-three Taiwan under-20 national futsal players were monitored for training intensity during 18 training camps. They were divided into two groups: selected (*n* = 14) and non-selected (*n* = 17) players. The physical assessments included the following measures: body mass, distance covered at Yo-Yo intermittent recovery test Level 1, final velocity at 30-15 Intermittent Fitness Test (30-15 IFT), standing long jump, maximum heart rate (HR), and 1-min sit-up. The training intensity was monitored using the rate of perceived exertion (RPE), HR at different intensity zones, and locomotor demands measured at different speed thresholds. The results revealed that the selected players were significantly faster in the 15-m sprint with ball (*p* = 0.001) and 30-m sprint (*p* = 0.001). Additionally, the selected players presented significantly greater HR_average_ and time spent above 90% maximum HR during the three-to-six-day training camps (*p* < 0.05) compared to the non-selected players. Interestingly, the NS demonstrated a greater number of sprints during the training camps (*p* = 0.001), while the selected players presented greater distance/minute and average speed (*p* = 0.001). A regression analysis showed that the distance/minute and average speed was a significant predictor of maximum HR in the selected players. As conclusions, the physical fitness outcomes are different between the selected and non-selected national futsal players. The selected players spent more time in high intensity HR demands in training sessions.

## 1. Introduction

Futsal is an invasion team sport in which the effort is intermittent, varying from low-to-moderate locomotor demands to very-high to all-out actions [1]. The distances covered during male futsal matches may vary between 3046 m in under-17 players [2] and 4300 [3] meters; from those, 571 [3] to 1105 [4] meters can be attained at high-intensity running, while sprinting may vary between 106 [4] and 348 [3] meters by professional players. The intermittence on locomotor demands also demonstrates variations in physiological responses. As an example, the oxygen uptake during the match can be, on average, around 48 mL/Kg/min, with a particular aspect that 46% of the playing time is spent above 80% of the maximal oxygen uptake in professional players [5]. As an intermittent exercise, the values of blood lactate in professional players can vary a great deal depending on the type of analysis since some of the reports present average values between 4.2 and 4.8 mmol/L [6] and others between 14.9 and 15 mmol/L [7]. As for heart rate responses, the values for the average of a match can be around or above 85% maximal heart rate (HR) in professional players [1].

Players must have specific physiological and neuromuscular adaptations to sustain the efforts of the match. An adult male futsal player may present values of maximal oxygen uptake between 49 [8] and 63 [9] mL/Kg/min, maximal heart rate between 184 [10] and 198 [11] beats per minute, and cover, on average, between 1160 [12] and 1507 [13] meters in the Yo-Yo Intermittent Recovery Test level 1 (YYIRT). Regarding vertical jump ability, male futsal players may present average values between 36.6 [14] and 50.4 [15] centimeters in the countermovement jump. Considering fat mass, male futsal players may vary between 10 [16] and 15% [17,18]. 

Although the above-described are the typical values for male futsal players, the competitive level can demonstrate differences in the physical fitness. In the case of aerobic fitness, elite players seem to have significant greater maximal oxygen uptake than sub-elite (62.9 vs. 55.2 mL/Kg/min, respectively) [9]. Moreover, significant differences were found between elite and sub-elite considering the performance achieved in the Futsal Intermittent Endurance Test (1378 vs. 1018 m, respectively) [19]. Considering body composition, elite players seems to have greater lean mass in dominant and non-dominant legs than lower-level players [20]. However, regarding fat mass, no significant differences seem to be found between elite and non-elite, or professional and semi-professional players [20,21]. 

In the context of national teams, coaches are often exposed to selecting situations in which the overall performance of a player may play a role in the ultimate decision of keeping the player or exclusion from the final list of selected players. Comparisons between selected and non-selected players have been performed in soccer, with the evidence suggesting that the selected players tend to be taller [22] and present significantly greater lean body mass [23], jumping performance [23], power [23], speed [22,23], and repeated sprint ability [22]. Although an interesting topic, this evidence is absent in futsal as far as we know. Considering that selected players are possibly in better shape than non-selected players, this could possibly translate to how they sustain efforts in training scenarios.

As an example, significant very large correlations were found between training intensities and the physical fitness presented in the YYIRT, while large correlations were found between training intensities and maximal oxygen uptake presented by high-level futsal players [24]. Following this evidence, a study also revealed a significant correlation between maximal oxygen uptake and overall training intensities accumulated over a period of four weeks [25].

Since physical fitness can be different between selected and non-selected players and considering that physical fitness seems to be related with the training intensities attained by players, it is relevant to identify if there are differences between selected and NS players in training demands occurring in training scenarios. Considering the absence of evidence regarding this matter in the futsal, and especially in national teams, our aim for the current study was two-fold: (i) analyze the variations in physical fitness and performance profiles of the selected and non-selected in under-20 male national futsal players; and (ii) analyze the variations in training intensity monitored during training camps. We hypothesized better physical fitness and performances for the selected players than the non-selected. Variations in the physical performance and locomotion profile with different training camp durations were also expected. 

## 2. Materials and Methods

### 2.1. Study Design

A cohort study design was followed.

### 2.2. Setting

Eighteen training camps (TC) of an under-20 male national futsal team were monitored over the years 2018 and 2019 (Table 1). The HR responses, rate of perceived exertion (RPE), and locomotor demands were monitored in all training sessions and matches occurring during the TC. Physical fitness assessments occurred twice over the two years. The final selection of players occurred at the end of the last TC in 2019. The physical fitness assessments were reported to the head coach as reference of physical level of players. The monitoring records in relation to training and match status were solely used by the sport trainers. The selection was exclusively responsible from the staff team with no influence of researchers that merely described those were selected and those not. Based on this final selection, those not selected were classified as non-selected players retrospectively (across the 18 TC and for the first round of assessment occurring in 2018), and the same for those selected.

### 2.3. Participants

Thirty-three Taiwan male futsal players were observed during the two-year period. At the beginning of the study, the average of age was 17.9 ± 0.8 years old, while, in the end of the period, the average was 18.4 ± 1.1 years old. Considering the last notification of selection of players, fourteen players were included in the list of selected players and the remaining seventeen were classified as non-selected. It is important to mention that, over the TC, the selected players were more regular in participating in most than non-selected. Eligibility criteria for being included in the current research were defined such that (i) players must be part of at least one TC; (ii) players must have had at least one physical fitness assessment during the observation period. Players were informed about the study design and protocol, risks, and benefits. The research followed the ethical standards for study in humans and was approved by a local ethical committee with the code UT-IRB-2018-068.

### 2.4. Physical Fitness Assessment Procedures

In the beginning of training session and after the initial warm-up, the physical fitness assessment was conducted during the first day afternoon of domestic training camps in 2018-TC1 (7 October 2018) and 2019-TC1 (15 June 2019). The players travelled from the home clubs/schools and arrived at the training center on the registration day of training camps. The players were required to perform the physical fitness assessment after the orientation of training camps delivered by the head coach. The players started with 10 min self-selected warm up activities supervised with the team sport trainer. Afterwards, the players were instructed to conduct anthropometry and body composition assessment, followed by standing long jump, one-minute sit up, and YYIRT level 1 in 2018-TC1. The testing protocol was set as following order in 2019-TC1: anthropometry and body composition assessment, Standing long jump (SLJ), Countermovement jump (CMJ), 15- and 30-m sprint (15 m Sprint and 30 m Sprint), 15 m sprint with ball (15 m SB), 1 min sit-up (1MSU), and 30–15 intermittent fitness test (30–15 IFT). The resting interval between the assessments was 5 min. The players were allowed to intake hydration throughout the physical fitness assessments. All assessments were conducted in an indoor sports complex hall with central-air condition.

#### 2.4.1. Anthropometry and Body Composition

The anthropometric measures of height and body weight were determined by a portable stadiometer (Seca 213, SECA, Hamburg, Germany) and a portable weight scale (Xyfwt382, Teco, Taiwan), respectively. The team sports trainers used a skin folder (Lange Skinfolder Caliper, Beta Technology, Cambridge, MD, USA) to quantify four locations of skinfold thickness. Subsequently, the percentage of body fat was determined by using Faulkner’s formula [26] as 5.783 + 0.153 × (the sum of triceps, subscapular, suprailiac, abdominal skinfolds)/100.

#### 2.4.2. One-Minute Sit Up

The one-minute full sit-up test (1MSU) was implemented for assessing abdominal strength and endurance [27]. The participants were instructed to lie on foam floor mats. One teammate held the participant’s ankle joint to stabilize the lower limbs’ positions. The participants were required to maintain knee position at 90 degrees with hands placed on the ears. The participants started with lying position and sit up when their elbows touched to the knees and then back to the starting position. The participants were encouraged to engage the endurance performance as much as possible in one minute. The total number of successful performances was recorded.

#### 2.4.3. Standing Long Jump

The standing long jump (SLJ) was implemented to assess maximal jump performance of the futsal players [27,28]. Participants positioned their feet immediately before the starting line and were instructed to perform a fast downward movement followed by a maximal effort horizontal jump. After landing (bilateral), the closer feet to the starting line were registered to measure the distance of the jump. The measurement was taken from the heel using a metric tape in centimeters. Participants performed a familiarization trial and three assessments interspaced by 3 min rest. The longest jump (meters) was considered for further data treatment.

#### 2.4.4. The Yo-Yo Intermittent Recovery Test—Level 1

The YYIRT—level 1 was implemented to measure the aerobic fitness of participants. We have followed the original protocol [29]. The test consists of to execute 2 × 20-m, following a walking recovery of 10-s. The test starts with a pace of 10 km/h (imposed by an audio beep), and progressively increases by 0.5 km/h until the player achieves the exhaustion. Four bouts 2 × 20-m are performed at 10–13 km/h, seven bouts are performed at 13.5–14 km/h, and, after that, eight bouts are performed for each stage [29]. The test was conducted in an indoor futsal court. The end of the tests was considered every time a player did not attain the pace or not reached the supposed line at the beep two consecutive times. The total distance completed during the test was considered as the main outcome.

#### 2.4.5. Countermovement Jump (CMJ)

The players performed the hands-on-waist CMJ [30] with feed shoulder-width apart on flat flood. All players were given two preliminary trials for practice. Afterwards, the players conducted 3 trials with 1 min resting interval. The height of CMJ performance was assessed by My jump 2 smartphone application [31]. The position of smartphone was set at a 4 m distance with level of recording at 1 m height. The best result was used for data analysis.

#### 2.4.6. 15- and 30-Meters Sprint (15 m Sprint and 30 m Sprint)

The sprint performance at 15-m and 30-m was assessed to evaluate individual maximal speed during linear sprint. An adhesive tape was marked 30 cm behind the starting line. The players were asked to perform 30-m sprint from a split stance in 3 times. Resting interval was set as 2 min. Two experienced assistant coaches used stop watches (Seiko, S056, Tokyo, Japan) to record the sprint time at 15-m and 30-m positions once the players crossed over the recording lines. The assistant coach recorded at 30-m position gave instruction to the players to sprints. The best result was used for data analysis.

#### 2.4.7. 15-Meters Sprint with Ball (15 m SB)

The testing preparation of 15 m SB is same as the 15 m Sprint and 30 m Sprint test. The players were instructed to dribble the ball in a straight line at maximal speed level. All the players performed 2 times with 2 min resting interval. The assistant coach recorded at 15-m position and gave instruction to the players to sprints. The best result was used for data analysis.

#### 2.4.8. 30-15 Intermittent Fitness Test (30-15 IFT)

The 30-15 IFT was implemented with the adjusted version for indoor court [32]. The tests consist of executing 30-s of running, interspaced by a 15-s period of recovery. The test starts at a pace of 8 km/h and increases 0.5 km/h at each new stage (30-s running). An audio beep governs the pace of players. The test ends in the moment of the player’s exhaustion or if the players are unable to reach the expected line at the time of the beep. The main outcome extracted is the final velocity completed at 30-15 IFT.

### 2.5. Monitoring Procedures

To monitor the players’ perceived and physical loads of training status, RPE, HR responses, and locomotion profiles were recorded during the training sessions. The RPE and HR recorded were used to quantify the internal load of training sessions, while the locomotion profiles were used to evaluate the external load of training sessions.

#### 2.5.1. Rate of Perceived Exertion

The CR-10 Borg’s scale was used to assess the player’s perception effort regarding each training session [33]. The CR-10 Borg’s scale consists in an ordinal scale in which 0 means nothing to all and 10 means very-very strong effort regarding the question “how intense was this training session?”. The players were allowed to provide a score in entire number or 0.5 (example: 5.5). The players were previously familiarized with the scale. After that, after 20–30 min of end of training session, the scores were collected individually. The score provided was the main outcome extracted. Moreover, the time of the session (minutes) was registered and considered for the calculus of session-RPE (sRPE), which represents the multiplication of time of the session by the RPE score provided [34]. The sRPE measured in arbitrary units was also collected per player as main outcome.

#### 2.5.2. Heart Rate Responses

The HR responses were collected using a heart rate sensor (Polar Team Pro GPS, Polar Electro, Kempele, Finland). Records of HR beats were taken in each second. The maximal HR (HR_max_) was also considered by adding the peak HR detected during the YYIRT. This was used to determine the heart rate zones and the time spent in each of them: (i) Zone 1 (Z1; 51–60%HR_max_); (ii) Zone 2 (Z2; 61–70%HR_max_); (iii) Zone 3 (Z3; 71–80%HR_max_); (iv) Zone 4 (Z4; 81–90%HR_max_); and (v) Zone 5 (Z5; 91–100%HR_max_). The main outcomes extracted for each training session were: (i) %HR_average_; (ii) %HR_max_; and (iii) time in HRZ5.

#### 2.5.3. Locomotor Demands

The locomotor demands were monitored using a GPS with a sampling frequency of 10 Hz (Polar Team Pro GPS, Polar Electro, Kempele, Finland). The GPS was previously confirmed for accuracy and reliability to collect measures of total distance covered and distances covered at different speed thresholds [35]. The following measures were extracted for each training session: (i) dist/min (average distance covered per minute); (ii) maximum speed (MS) (peak speed registered in the session); (iii) Average speed (AS); (iv) Sprint (number of sprints >25 km/h).

### 2.6. Statistical Procedures

The statistical analyses were carried out by using the software Statistica (version 13.1; Statsoft, Inc., Tulsa, OK, USA). Descriptive statistics were represented as mean ± SD. Tests of normal distribution and homogeneity (Kolmogorov–Smirnov and Levene’s, respectively) were conducted on all data before analysis. Paired sample *t*-test was used for determining differences (selected vs non-selected players). Cohen *d* was the effect size indicator. To interpret the magnitude of the effect size, we adopted the following criteria: *d* = 0.20, small; *d* = 0.50, medium; and *d* = 0.80, large. A Pearson correlation coefficient *r* was used to examine the relationship between values of physical fitness assessment [maximal oxygen uptake (VO2_max_) and HR_max_ obtained in YYIRT1, 30-15 IFT, 1MSU, SLJ, CMJ, 15 m Sprint, 30 m Sprint, and 15 m SB and different performance variables recollected in different training camps (sRPE), HR responses [(HR_average_ (HR_max_ %), and HR time in zone 5. HR (TZ5 HR)], and locomotor demands [Distance/minute (dis/min), MS, AS, and Sprint number]. To interpret the magnitude of these correlations we adopted the following criteria: *r* ≤ 0.1, trivial; 0.1 < *r* ≤ 0.3, small; 0.3 < *r* ≤ 0.5, moderate; 0.5 < *r* ≤ 0.7, large; 0.7 < *r* ≤ 0.9, very large; and *r* > 0.9, almost perfect. Posteriorly, Regression analysis was used to identify which values of physical fitness can better explain the sRPE, heart rate responses and locomotor demands. The magnitude of R2 was interpreted as follows: >0.02, small; >0.13, medium; >0.23, large.

## 3. Results

### 3.1. Anthropometric Characteristics and Performance Variables

Descriptive statistics were calculated for each variable (Table 2). Paired measures *t*-test with anthropometric characteristics (age; height, weight, % F and % M) and performance variables were collected (sRPE, HR_average,_ TZ5. HR, dis/min, MS, AS, and S) in the different TCs and calculated as a mean (TC. 2 days, TC 3 days, TC. 4 days, TC. 5 days, TC. 6 days, TC. 7 days, TC. 9 days, and TC. 10 days) to observe the differences between the selected and non-selected players (See Table 2 for more information).

### 3.2. Performance Variables and Locomotor Demands

Paired measures *t*-test with performance variables (sRPE, HR_average_, VO2_max_, HR_max_, and TZ5 HR), locomotor demands (dis/min, MS, AS, and sprint number) recollected in different training camps (TC. 2 days; TC. 3 days; TC. 4 days, and TC. 5 days) were realized to show the difference between selected and non-selected players (Table 3). Posteriorly, the same analysis was performed but in this case with TC (TC. 6 days; TC. 7 days; TC. 8 days, and TC. 9 days) (Table 4).

Complementarily, data from both seasons were used to calculate general TC data (TC from two to ten days). Then, a paired measures *t*-test was used to compare selected and non-selected players regarding performance variables and locomotor demands (Table 5).

Posteriorly, a correlation analysis was performed between the performance variables (sRPE, HR_average_, and TZ5 HR), locomotor demands (dis/min, MS, AS, and Sprint number), and physical fitness assessment (YYIRT1, VO2_max_, HR_max_, 30-15, 1MSU, SLJ, CMJ, 15 m Sprint, 15 m SB, and 30 m Sprint) for the selected players (see Table 6).

Table 7 presents a multilinear regression analysis that was performed to verify which physical fitness assessment (HR_max_, 1 MSU, 30-15 IFT, and VO2_max_) agreement with the correlation analysis could be used to better explain the performance variables and locomotor demands (Dist/min, AS, and TZ5 HR).

At this point, the same correlation analysis was performed between performance variables (sRPE, HR_average_, HR_max_ %, and TZ5 HR), locomotor demands (dis/min, MS, AS, and Sprint), and physical fitness assessment (YYIRT1, VO2_max_, 30–15 IFT, 1MSU, SLJ, CMJ, 15 m Sprint, 15 m SB, and 30 m Sprint) but, in this case, for the non-selected players (See Table 8, for more information).

Lastly, a new multilinear regression analysis was presented in Table 9 to verify which physical fitness assessment (SLJ, 15 m SB, and 30 m Sprint) agreement with the correlation analysis could be used to better explain the performance variables and locomotor demands (sRPE, HR_average_, Dist/min, AS, and TZ5 HR).

## 4. Discussion

The present study aimed to analyze the variations in the physical fitness of the selected and non-selected under-20 male national futsal players and the variations in training intensity monitored in the training camps. The main findings showed differences in the performance variables, namely 15 m Sprint and 30 m Sprint, where lower values were found for the selected, which means better performances for the selected than non-selected. Considering all the tests included for analysis, the data from the present study seem to support that the ability to perform linear sprints of 15 and 30 m is the most important when selecting players. In fact, sprints of 15 m Sprint [22] and 30 m Sprint [23] were previously reported as determinant to select under-17 soccer players. Additionally, a recent systematic review has shown that elite futsal players cover greater total distance with higher intensities and perform a greater number of sprints during match-play when compared to sub-elite players [1], which reinforces our findings. However, a study conducted in female elite and sub-elite futsal players presented contrary results by showing no differences between groups [36], which suggests that more studies are required to confirm the present results.

Hence, the findings of our study suggest that better 30 m sprint may contribute to an advantage for selected players in order to apply their technical skills and motor abilities for better ball manipulation and possession [36].

Furthermore, when analyzing the different TCs, there were more differences between the selected versus non-selected players. In general, higher values were found for the selected with the exceptions of sprint (TC, 3–10 days) and maximum speed (TC, 5–9 days), dist/min (TC, 9 and 10 days), and average speed (TC, 9 days). The exceptions could be explained by some specific exercises/tasks performed in TC and the previous knowledge about those specific exercises/tasks that were not considered for this study, but it could explain the results as previously reported [37]. We speculate that the selected players could better manage their effort while avoiding sprints during TC compared to the non-selected players. Even so, the overall results tended to show better performance and locomotor demands for the selected than non-selected players, which is supported by previous studies that showed similar results for selected soccer players [22,23] when compared to non-selected, or top-level players [9,19] when compared with lower levels.

Moreover, when analyzing all the TCs together, all the performance and locomotor demands were significant different. Generally, the selected players showed lower sRPE and higher HR_average_ and duration and TZ5 HR. Regarding the locomotor demands, the selected players showed higher distance/min, average speed, but lower maximum speed and number of sprints.

The results are partially supported by a previous systematic review that showed that elite futsal players covered greater total distance with higher intensities and performed a greater number of sprints during match-play when compared to sub-elite players [1]. We believe that the data from under-20 players and from TC would reveal such similarities as in the present study, but more research is needed to confirm the results.

Meanwhile, the sRPE showed an opposite result with lower values for the selected players. There are some justifications for this finding. First, some studies mentioned that RPE may be a physiological and volatile construct because it can dissociate the physiological process from the psychological mechanisms and, consequently, could not provide all the sensations of the effort experience [38]. Second, the RPE was collected 30 min after each session, which does reveal the variations within sessions [39]. Third, there is a possibility that the selected players, due to the higher training adaptations, may interpret lower RPE values when compared to the non-selected players.

To the best knowledge of the authors, this seems to be the first study to include an analysis based on different TC periods, which seems to provide greater knowledge for coaches and their staff. However, it is important to acknowledge how to conduct the statistical analysis. As observed in the present study, different approaches provided different results and, for this reason, we suggest that future studies could replicate such a design to amplify the knowledge in this field and, at the same time, to provide relevant information for coaches.

Additionally, the correlation analyses for the selected players showed associations between TZ5 HR with 1M.SU, 30–15 IFT, and VO2_max_. Thus, a regression analysis was conducted in an attempt to explain which variable could influence the performance and locomotor demands more, but it fails to explain any result. Therefore, future studies could use other variables for the analysis. On the other hand, the correlation analyses for the non-selected players showed several associations (see Table 8), and, thus, another regression analysis was conducted. It showed relevant results for coaches and their staff. The main results showed that the 30 m S test could be explained by sRPE, HR_average_, HR_max_, dist/min, MS, and AS between 38 and 48%, while the 30 m Sprint was one of the main variables that showed a significant difference between the groups analysed in this study, and it seems that this test is representative of a maximal test and, consequently, presents several higher values for physiological and locomotor demands, which was also shown by the 15 m SB and could also be explained 49% by the TZ5 HR. The importance of agility while dibbling a ball is extremely important not only to better manipulate the ball but also for possession and better changes in direction [40].

An interesting finding was that this regression only explained the results for the non-selected players, which suggests the necessity of more studies to confirm the present results.

This study presents some limitations that should be stated. Firstly, the small sample size does not allow the generalization of the results. Secondly, the selected players were only defined in the end of the last TC, which may have influenced the results. Thirdly, information about the exercise/tasks performed during TC was not taken into consideration, which may had provided more and specific knowledge. Fourthly, the players’’ physical and locomotor performance during the TC was not reported to the coaching staff. The potential bias for the final selection may be limited by the optimal physical performance reported in the physical fitness assessments. Fifthly, the number of training days and called-up players varied from TC to TC due to logistics issues from the federation, players selection by coaching staff, and conflicts regarding the home team’s schedule. Finally, not all the tests were performed in the two assessments. Thus, we suggest that future studies could be conducted avoiding such limitations. Additionally, they should replicate such a design in other categories and women futsal players.

At last, there is a possibility that other variables, such as shooting, passing, dribbling, ball control, and tactical skills, could be used to identify and distinguish selected from non-selected players, as previously reported [19,22,23]. For that reason, we suggest that future studies should considered performance, locomotor, and skill variables for comprehensive analysis of selected and non-selected futsal players.

## 5. Conclusions

The selected players had superior performances in the 15 m Sprint and 30 m Sprint compared to the non-selected players. Additionally, the selected players had optimal performance variables and locomotor demands compared to the non-selected players. Moreover, it was not feasible to establish a relationship between the physical fitness tests and performance variables/locomotor demands for the selected players, while it was possible to observe several associations between the physical fitness tests (SLJ, 15 m SB, and 30 m Sprint) and performance variables/locomotor demands for the non-selected players. Coaches and their staff could consider the information of the present study for identifying selected players. Meanwhile, data from this study would help coaches and their staff to improve physical fitness tests for non-selected players.

## Figures and Tables

**Table 1:** Timeline of the study.

Training Camps	2018-TC1	2018-IT	2018-TC2	2018-TC3	2018-TC4	2018-TC5	2018-TC6	2018-OS	2018-Q	2019-TC1	2019-TC2	2019-TC3	2019-OS1	2019-IT	2019-TC4	2019-TC5	2019-OS2	2019-F
Starting day	09 July 2018	28 July 2018	01 September 2018	21 September 2018	05 October 2018	18 October 2018	05 November 2018	19 November 2018	27 November 2018	18 February 2019	04 March 2019	25 March 2019	07 April 2019	20 April 2019	10 May 2019	27 May 2019	01 June 2019	12 June 2019
Ending day	15 July 2018	02 August 2018	07 September 2018	27 September 2018	11 October 2018	24 October 2018	11 November 2018	22 November 2018	02 December 2018	22 February 2019	09 March 2019	29 March 2019	11 April 2019	28 April 2019	12 May 2019	29 May 2019	09 June 2019	15 June 2019
TC days (*n*)	7	5	7	7	7	7	4	4	6	5	6	5	6	9	3	2	9	4
Fitness assessment	X									X								
Selected players (*n*)	9	9	11	11	12	12	12	12	11	11	11	12	14	14	14	14	14	14
Non-selected players (*n*)	9	3	11	6	7	6	7	8	3	10	9	10	5	2	4	0	0	0

F: final competition; IT: invitation tournament; OS: overseas training camp; Q: qualifier competition; TC: training camp.

**Table 2:** Anthropometric characteristics and performance variables (mean ± SD).

	Season 2018–2019			Season 2019–2020		
	Selected	Non-Selected	*p|d*	Selected	Non-Selected	*p|d*
Anthropometric characteristics						
Age (yrs)	18.07 ± 0.83	17.75 ± 0.87	*p* = 0.39*|d* = 0.38	18.69 ± 0.85	18.00 ± 1.21	*p* = 0.13*|d* = 0.66
Height (cm)	171 ± 8	173 ± 6	*p* = 0.53*|d* = −0.34	173 ± 6	174 ± 6	*p* = 0.94*|d* = −0.11
Weight (kg)	65.98 ± 12.10	65.39 ± 6.40	*p* = 0.98*|d* = 0.06	66.95 ± 11.24	64.28 ± 6.25	*p* = 0.65*|d* = 0.29
% Fat	12.68 ± 3.29	12.18 ± 2.58	*p* = 0.73*|d* = 0.17	12.38 ± 1.76	12.61 ± 2.20	*p* = 0.51*|d* = −0.12
% Muscle	44.11 ± 2.99	44.44 ± 3.29	*p* = 0.69*|d* = −0.10	43.57 ± 1.66	42.95 ± 2.00	*p* = 0.64*|d* = 0.34
Performance variables						
YYIR1 (m)	1793.85 ± 406.56	1876.00 ± 348.94	*p* = 0.73*|d* = −0.22	-	-	-
30–15 IFT (km/h)	-	-	-	19.64 ± 1.48	20.00 ± 1.00	*p* = 0.26*|d* = −0.29
VO2_max_ (mL/kg/min)	51.47 ± 3.42	52.16 ± 2.93	*p* = 0.33*|d* = −0.22	51.48 ± 3.31	52.16 ± 2.31	*p* = 0.33*|d* = −0.24
1MSU (Total)	54.08 ± 6.02	53.80 ± 8.95	*p* = 0.79*|d* = 0.04	58.56 ± 5.15	55.90 ± 4.95	*p* = 0.39*|d* = 0.53
SLJ (cm)	230 ± 18	229 ± 9	*p* = 0.80*|d* = 0.08	246 ± 17	237 ± 10	*p* = 0.28*|d* = 0.61
CMJ (cm)	-	-	*-*	51.50 ± 5.73	46.24 ± 6.03	*p* = 0.17*|d* = 0.89
15 m Sprint (s)	-	-	*-*	2.83 ± 0.11	2.94 ± 0.08	*p* = 0.001 **|d = −1.15
15 m SB (s)	-	-	*-*	3.06 ± 0.12	3.15 ± 0.15	*p* = 0.24*|d* = −0.73
30 m Sprint (s)	-	-	*-*	4.69 ± 0.15	4.80 ± 0.12	*p* = 0.001 **|d = −0.81

Note: Yrs: years; YYIR1: Yo-Yo intermittent recovery test level 1; 30-15 IFT: final velocity at 30-15 intermittent fitness test; VO2_max_: maximal oxygen uptake; SLJ: standing long jump; CMJ: countermovement jump; SB: sprint with ball. ** denotes significance at *p* < 0.01.

**Table 3:** Performance variables (sRPE, HR_average_, VO2_max_, and TZ5 HR) and locomotor demands (dis/min, maximal speed, average speed, and sprint number) recollected in different training camps (TC. 2 days, TC. 3 days, TC. 4 days, and TC. 5 days).

Variables		TC. 2 Days			TC. 3 Days			TC. 4 Days			TC. 5 Days		
		Selected	Non-Selected	*p|d*	Selected	Non-Selected	*p|d*	Selected	Non-Selected	*p|d*	Selected	Non-Selected	*p|d*
Performance variables	sRPE (AU)	1262.88 ± 376.59	1195.00 ± 311.13	*p* = 0.12|*d* = 0.19	725.04 ± 353.61	558.80 ± 268.91	*p* = 0.07|*d* = 0.53	691.37 ± 348.70	767.59 ± 317.08	*p* = 0.16|*d* = −0.23	714.93 ± 408.99	776.54 ± 397.95	*p* = 0.03 *|*d* = −0.15
	HR_average_ (%)	67.85 ± 7.59	67.50 ± 7.78	*p* = 0.14|*d* = 0.04	67.77 ± 9.64	68.00 ± 6.24	*p* = 0.56|*d* = −0.03	69.26 ± 4.77	63.79 ± 8.17	*p* = 0.002 **|*d* = 0.82	68.17 ± 7.54	64.10 ± 6.77	*p* = 0.001 **|*d* = 0.57
	TZ5 HR (min)	16.37 ± 12.21	23.59 ± 16.50	*p* = 0.82|*d* = −0.49	12.03 ± 13.48	11.34 ± 15.54	*p* = 0.18|*d* = 0.03	17.36 ± 12.20	11.33 ± 11.05	*p* = 0.005 **|*d* = 0.51	10.04 ± 9.45	06.00 ± 08.05	*p* = 0.001 **|*d* = 0.44
Locomotor demands	Dist/min (m/min)	37.64 ± 5.97	41.55 ± 0.64	*p* = 0.83|*d* = −0.92	41.41 ± 11.69	47.53 ± 6.86	*p* = 0.35|*d* = −0.64	40.23 ± 11.79	36.67 ± 15.02	*p* = 0.38 |*d* = 0.26	38.03 ± 8.77	37.96 ± 10.83	*p* = 0.23|*d* = 0.01
	MS (km/h)	27.59 ± 1.47	28.01 ± 1.36	*p* = 0.47|*d* = −0.29	25.01 ± 4.47	26.78 ± 0.89	*p* = 0.42|*d* = −0.55	26.80 ± 2.18	27.95 ± 4.31	*p* = 0.56 |*d* = −0.33	26.02 ± 2.71	27.81 ± 4.63	*p* = 0.001 **|*d* = −0.50
	AS (m/s)	2.38 ± 0.36	2.58 ± 0.15	*p* = 0.75|*d* = −0.71	2.51 ± 0.70	2.94 ± 0.45	*p* = 0.16|*d* = −0.75	2.44 ± 0.71	2.22 ± 0.90	*p* = 0.001 **|*d* = 0.27	2.34 ± 0.55	2.35 ± 0.64	*p* = 0.19|*d* = −0.01
	Sprints (*n*)	12.05 ± 7.09	23.50 ± 0.71	*p* = 0.11|*d* = −2.27	8.77 ± 6.52	32.50 ± 22.70	*p* = 0.003 **|*d* = −1.42	13.41 ± 13.63	20.29 ± 19.24	*p* = 0.05 * |*d* = −0.41	11.30 ± 10.10	26.66 ± 22.23	*p* = 0.001 **|*d* = −0.89

Note: sRPE: session rate of perceived exertion; AU: arbitrary units; HR: heart rate; TZ5: time in zone 5. Dis: distance; MS: maximum speed; AS: average speed; *n* = number. * Denotes significance at *p* < 0.05, and ** denotes significance at *p* < 0.01.

**Table 4:** Performance variables (sRPE, HR_average_, VO2_max_, and TZ5 HR) and locomotor demands (dis/min, maximal speed, average speed, and sprint number) recollected in different training camps (TC. 6 days, TC. 7 days, TC. 9 days, and TC. 10 days).

Variables		TC. 6 Days			TC. 7 Days			TC. 9 Days			TC. 10 Days		
		Selected	Non-Selected	*p|d*	Selected	Non-Selected	*p|d*	Selected	Non-Selected	*p|d*	Selected	Non-Selected	*p|d*
Performance variables	sRPE (AU)	650.67 ± 334.06	787.69 ± 334.49	*p* = 0.36|*d* = −0.41	965.73 ± 427.09	943.59 ± 413.77	*p* = 0.75|*d* = 0.05	571.42 ± 373.82	443.64 ± 311.13	*p* = 0.003 **|*d* = 0.42	575.07 ± 288.02	560.00 ± 177.77	*p* = 0.06|*d* = 0.06
	HR_average_ (%)	68.91 ± 7.65	62.93 ± 6.49	*p* = 0.001 **|*d* = 0.84	69.57 ± 6.93	66.99 ± 6.66	*p* = 0.001 **|*d* = 0.38	70.24 ± 5.55	70.13 ± 3.11	*p* = 0.59|*d* = 0.02	70.39 ± 6.28	75.63 ± 3.20	*p* = 0.29|*d* = −1.05
	TZ5 HR (min)	12.21 ± 10.47	07.33 ± 10.37	*p* = 0.005 **|*d* = 0.45	17.07 ± 15.12	12.39 ± 15.09	*p* = 0.01*|*d* = 0.29	07.48 ± 09.22	06.23 ± 06.25	*p* = 0.16|*d* = 0.18	12.52 ± 09.44	24.00 ± 08.23	*p* = 0.81|*d* = −1.23
Locomotor demands	Dist/min (m/min)	42.25 ± 12.27	41.71 ± 11.97	*p* = 0.08|*d* = 0.04	50.57 ± 14.96	50.16 ± 14.77	*p* = 0.58|*d* = 0.03	49.72 ± 10.00	58.75 ± 11.36	*p* = 0.003 **|*d* = −0.84	43.10 ± 10.15	54.70 ± 7.74	*p* = 0.01 *|*d* = −1.29
	MS (km/h)	27.55 ± 3.40	28.63 ± 3.67	*p* = 0.04 *|*d* = −0.31	27.03 ± 2.37	28.92 ± 4.73	*p* = 0.001 **|*d* = −0.51	27.21 ± 1.97	29.90 ± 2.95	*p* = 0.001 **|*d* = −1.07	28.07 ± 2.66	30.79 ± 2.82	*p* = 0.32|*d* = −0.99
	AS (m/s)	2.57 ± 0.74	2.53 ± 0.72	*p* = 0.07|*d* = 0.06	3.07 ± 0.89	3.05 ± 0.92	*p* = 0.59|*d* = 0.02	3.02 ± 0.57	3.53 ± 0.67	*p* = 0.002 **|*d* = −0.82	2.61 ± 0.61	3.30 ± 0.47	*p* = 0.01|*d* = −1.26
	Sprints (*n*)	11.30 ± 8.48	30.86 ± 21.83	*p* = 0.001 **|*d* = −1.18	22.98 ± 20.71	27.72 ± 22.52	*p* = 0.01 *|*d* = −0.22	10.59 ± 7.34	32.47 ± 15.25	*p* = 0.002 **|*d* = −1.83	11.88 ± 7.67	30.13 ± 7.68	*p* = 0.01 *|*d* = −0.89

Note: sRPE: session rate of perceived exertion; AU: arbitrary units; HR: heart rate; TZ5: time in zone 5. Dis: distance; MS: maximum speed; AS: average speed; *n* = number. * Denotes significance at *p* < 0.05, and ** denotes significance at *p* < 0.01.

**Table 5:** Performance variables (sRPE, HR_average_, VO2_max_, and TZ5 HR) and locomotor demands (dis/min, MS, AS, and S) recollected in different training camps (TC. 2 days, TC. 3 days, TC. 4 days, TC. 5 days, TC. 6 days, TC. 7 days, TC. 9 days, and TC. 10 days) recorded with overall data.

Variables		Selected	Non-Selected	*p|d*
Performance variables	sRPE (AU)	812.46 ± 417.35	841.44 ± 399.72	*p* = 0.005 **|*d* = −0.07
	HR_average_ (%)	69.21 ± 7.08	65.51 ± 6.99	*p* = 0.001 **|*d* = 0.53
	TZ5 HR (min)	13.50 ± 12.46	09.50 ± 12.39	*p* = 0.004 **|*d* = 0.31
Locomotor demands	Distance/min (m/min)	44.86 ± 13.33	44.75 ± 14.20	*p* = 0.001 **|*d* = 0.01
	MS (km/h)	27.06 ± 2.82	28.53 ± 4.33	*p* = 0.001 **|*d* = −0.40
	AS (m/s)	2.73 ± 0.79	2.72 ± 0.86	*p* = 0.001 **|*d* = 0.01
	Sprints (*n*)	15.47 ± 15.29	28.01 ± 21.65	*p* = 0.001 **|*d* = −0.67

Note: sRPE: session rate of perceived exertion; HR: heart rate; TZ5: time in zone 5. Dis: distance; *n* = number; MS: maximum speed; AS: average speed. ** denotes significance at *p* < 0.01.

**Table 6:** Correlation between performance variables, locomotor demands, and physical fitness assessment for selected futsal players.

			sRPE (AU)	HR_average_ (%)	HR_max_ (%)	TZ5. HR (min)	Dist/min (m/min)	MS (km/h)	AS (km/h)	Sprint (*n*)
Season 2018–2019	YYIR1 (m)	*r*	−0.16	−0.27	−0.26	−0.57	0.014	−0.15	0.01	0.43
		*p*	*p* = 0.75	*p* = 0.59	*p* = 0.60	*p* = 0.23	*p* = 0.97	*p* = 0.77	*p* = 0.97	*p* = 0.39
	VO2_max_ (mL/kg/min)	*r*	−0.16	−0.27	−0.26	−0.57	0.014	−0.15	0.018	0.43
		*p*	*p* = 0.75	*p* = 0.59	*p* = 0.60	*p* = 0.23	*p* = 0.97	*p* = 0.77	*p* = 0.97	*p* = 0.39
	1MSU (*n*)	*r*	−0.64	−0.67	−0.62	−0.91	−0.43	−0.51	−0.43	0.31
		*p*	*p* = 0.16	*p* = 0.14	*p* = 0.18	*p* = 0.01 *	*p* = 0.38	*p* = 0.30	*p* = 0.38	*p* = 0.54
	SLJ (cm)	*r*	0.53	0.36	0.37	0.30	0.25	0.26	0.25	−0.52
		*p*	*p* = 0.27	*p* = 0.48	*p* = 0.45	*p* = 0.55	*p* = 0.62	*p* = 0.61	*p* = 0.62	*p* = 0.28
Season 2019–2020	CMJ (cm)	*r*	0.69	0.46	0.45	0.44	0.35	0.32	0.35	−0.57
		*p*	*p* = 0.12	*p* = 0.35	*p* = 0.36	*p* = 0.37	*p* = 0.49	*p* = 0.53	*p* = 0.49	*p* = 0.23
	15 m Sprint (s)	*r*	−0.49	−0.51	−0.51	−0.78	−0.36	−0.36	−0.36	0.58
		*p*	*p* = 0.32	*p* = 0.29	*p* = 0.29	*p* = 0.06	*p* = 0.47	*p* = 0.47	*p* = 0.47	*p* = 0.22
	15 m SB (s)	*r*	−0.18	−0.40	−0.47	−0.17	−0.59	−0.56	−0.58	−0.58
		*p*	*p* = 0.71	*p* = 0.42	*p* = 0.34	*p* = 0.73	*p* = 0.21	*p* = 0.24	*p* = 0.21	*p* = 0.22
	30 m Sprint (s)	*r*	−0.72	−0.56	−0.59	−0.39	−0.70	−0.54	−0.70	0.14
		*p*	*p* = 0.10	*p* = 0.23	*p* = 0.21	*p* = 0.43	*p* = 0.11	*p* = 0.26	*p* = 0.12	*p* = 0.78
	30–15 IFT (km/h)	*r*	−0.57	−0.57	−0.54	−0.85	−0.32	−0.39	−0.32	0.53
		*p*	*p* = 0.23	*p* = 0.23	*p* = 0.26	*p* = 0.03 *	*p* = 0.53	*p* = 0.43	*p* = 0.53	*p* = 0.27
	VO2_max_ (mL/kg/min)	*r*	−0.58	−0.58	−0.55	−0.82	−0.32	−0.40	−0.32	0.54
		*p*	*p* = 0.21	*p* = 0.22	*p* = 0.25	*p* = 0.04 *	*p* = 0.52	*p* = 0.42	*p* = 0.52	*p* = 0.26
	1MSU (*n*)	*r*	−0.21	−0.35	−0.30	−0.68	−0.15	−0.23	−0.16	0.09
		*p*	*p* = 0.68	*p* = 0.48	*p* = 0.56	*p* = 0.13	*p* = 0.76	*p* = 0.64	*p* = 0.76	*p* = 0.85

Note: sRPE: session rate of perceived exertion; AU: arbitrary units; HR: heart rate; T5: time in zone 5. Dis: distance; MS: maximum speed; AS: average speed; YYIR1: Yo-Yo intermittent recovery test level 1; *n* = number; VO2_max_: maximal oxygen uptake; SLJ: standing long jump; CMJ: countermovement jump; SB: sprint with ball. * Denotes significance at *p* < 0.05.

**Table 7:** Values of regression analysis explaining physical fitness assessment on performance variables and locomotor demands for selected futsal players.

Variables		B	SE of b	R	R^2^	Adjusted R^2^	F	*p*
HR_max_	Dist/min	−0.60	0.28	0.60	0.36	0.28	4.58	0.06
	AS	−0.59	0.28	0.59	0.35	0.27	4.42	0.06
1 MSU	TZ5 HR	−0.56	0.27	0.56	0.31	0.24	4.18	0.07
30–15 IFT	TZ5 HR	−0.47	0.35	0.47	0.22	0.09	1.73	0.23
VO2_max_	TZ5 HR	−0.43	0.37	0.43	0.18	0.05	1.38	0.28

Note: HR: heart rate; 30-15 IFT: final velocity at 30-15 intermittent fitness test; TZ5: time at zone 5; AS: average speed; dist/min: distance/minute; 1MSU: 1 min sit-up; VO2_max_: maximal oxygen uptake; *interaction.

**Table 8:** Correlation between performance variables, locomotor demands, and physical fitness assessment for non-selected futsal players.

Variables			sRPE (AU)	HR_average_ (%)	HR_max_ (%)	TZ5. HR (min)	Dist/min (m/min)	MS (km/h)	AS (km/h)	Sprint (*n*)
Season 2018–2019	YYIR1 (m)	*r*	−0.13	−0.19	−0.10	−0.10	−0.27	−0.26	−0.26	−0.58
		*p*	*p* = 0.72	*p* = 0.61	*p* = 0.78	*p* = 0.78	*p* = 0.48	*p* = 0.48	*p* = 0.48	*p* = 0.10
	VO2_max_ (mL/kg/min)	*r*	−0.36	−0.23	−0.20	−0.37	−0.26	−0.27	−0.26	−0.10
		*p*	*p* = 0.33	*p* = 0.53	*p* = 0.59	*p* = 0.32	*p* = 0.48	*p* = 0.47	*p* = 0.49	*p* = 0.78
	1 MSU (*n*)	*r*	0.23	0.20	0.25	−0.45	0.18	0.15	0.18	0.02
		*p*	*p* = 0.55	*p* = 0.58	*p* = 0.51	*p* = 0.22	*p* = 0.63	*p* = 0.69	*p* = 0.63	*p* = 0.94
	SLJ (cm)	*r*	0.69	0.56	0.59	0.16	0.52	0.51	0.52	0.075
		*p*	*p* = 0.03 *	*p* = 0.10	*p* = 0.09	*p* = 0.66	*p* = 0.14	*p* = 0.15	*p* = 0.14	*p* = 0.84
Season 2019–2020	CMJ (cm)	*r*	0.01	−0.11	−0.14	−0.05	−0.20	−0.13	−0.208	−0.21
		*p*	*p* = 0.98	*p* = 0.76	*p* = 0.71	*p* = 0.89	*p* = 0.59	*p* = 0.72	*p* = 0.59	*p* = 0.58
	15 m Sprint (s)	*r*	−0.30	−0.32	−0.31	−0.49	−0.26	−0.30	−0.268	−0.09
		*p*	*p* = 0.42	*p* = 0.39	*p* = 0.41	*p* = 0.17	*p* = 0.48	*p* = 0.43	*p* = 0.48	*p* = 0.80
	15 m SB (s)	*r*	−0.47	−0.59	−0.53	−0.74	−0.48	−0.51	−0.48	−0.32
		*p*	*p* = 0.19	*p* = 0.09	*p* = 0.13	*p* = 0.02 *	*p* = 0.18	*p* = 0.15	*p* = 0.18	*p* = 0.39
	30 m Sprint (s)	*r*	−0.67	−0.74	−0.73	−0.42	−0.72	−0.74	−0.73	−0.57
		*p*	*p* = 0.04 *	*p* = 0.02 *	*p* = 0.02 *	*p* = 0.25	*p* = 0.02 *	*p* = 0.02 *	*p* = 0.02 *	*p* = 0.10
	30–15 IFT (km/h)	*r*	0.19	0.34	0.35	−0.037	0.44	0.40	0.44	0.59
		*p*	*p* = 0.61	*p* = 0.36	*p* = 0.34	*p* = 0.92	*p* = 0.22	*p* = 0.28	*p* = 0.22	*p* = 0.09
	VO2_max_ (mL/kg/min)	*r*	0.20	0.37	0.37	0.01	0.43	0.40	0.43	0.57
		*p*	*p* = 0.59	*p* = 0.32	*p* = 0.31	*p* = 0.98	*p* = 0.23	*p* = 0.28	*p* = 0.23	*p* = 0.10
	1MSU (*n*)	*r*	0.12	0.13	0.15	−0.46	0.14	0.11	0.14	0.19
		*p*	*p* = 0.75	*p* = 0.72	*p* = 0.69	*p* = 0.20	*p* = 0.70	*p* = 0.76	*p* = 0.71	*p* = 0.61

Note: sRPE: session rate of perceived exertion; AU: arbitrary units; HR: heart rate; T5: time in zone 5. Dis: distance; MS: maximum speed; 1MSU: 1 min sit-up; AS: average speed; YYIR1: Yo-Yo intermittent recovery test level 1; *n* = number; VO2_max_: maximal oxygen uptake; SLJ: standing long jump; CMJ: countermovement jump; SB: sprint with ball. * Denotes significance at *p* < 0.05.

**Table 9:** Values of regression analysis explaining physical fitness assessment on performance variables and locomotor demands for non-selected futsal players.

Variables		b	SE of b	R	R^2^	Adjusted R^2^	F	*p*
SLJ	sRPE	0.69	0.27	0.69	0.48	0.41	6.64	0.03 *
15 m SB	TZ5 HR	−0.74	0.25	0.74	0.56	0.49	8.96	0.02 *
30 m Sprint	sRPE	−0.67	0.27	0.67	0.46	0.38	5.98	0.04 *
	HR_average_	−0.74	0.25	0.74	0.55	0.48	8.65	0.02 *
	Dist/min	−0.73	0.25	0.73	0.53	0.46	7.98	0.02 *
	MS	−0.74	0.25	0.74	0.55	0.48	8.60	0.02 *
	AS	−0.74	0.25	0.74	0.55	0.48	8.60	0.02 *

Note: SLJ: standing long jump; sRPE: session rate of perceived exertion; HR: heart rate; Dist/min: distance/minute; MS: maximum speed; AS: average speed; 15 m SB: 15-m sprint with ball; TZ5: time at zone 5. * Denotes significance at *p* < 0.05.

## Data Availability

The data are available upon request to corresponding authors.

## References

[1] Spyrou K., Freitas T.T., Marín-Cascales E., Alcaraz P.E. (2020). Physical and physiological Match-play demands and player characteristics in futsal: A systematic review. Front. Psychol..

[2] Milanez V.F., Bueno M.J.D.O., Caetano F.G., Chierotti P., De Moraes S.M.F., Moura F.A. (2020). Relationship between number of substitutions, running performance and passing during under-17 and adult official futsal matches. Int. J. Perform. Anal. Sport.

[3] Barbero-Alvarez J.C., Soto V.M., Barbero-Alvarez V., Granda-Vera J. (2008). Match analysis and heart rate of futsal players during competition. J. Sports Sci..

[4] Doğramacı N.S., Watsford L.M. (2006). A comparison of two different methods for time-motion analysis in team sports. Int. J. Perform. Anal. Sport.

[5] Castagna C., D’Ottavio S., Granda Vera J., Barbero Alvarez J.C., D’Ottavio S., Vera J.G., Álvarez J.C.B. (2009). Match demands of professional Futsal: A case study. J. Sci. Med. Sport.

[6] Milioni F., Vieira L.H.P., Barbieri R.A., Zagatto A.M., Nordsborg N.B., Barbieri F.A., Dos-Santos J.W., Santiago P.R.P., Papoti M. (2016). Futsal match-related fatigue affects running performance and neuromuscular parameters but not finishing kick speed or accuracy. Front. Physiol..

[7] Bekris E., Gioldasis A., Gissis I., Katis A., Mitrousis I., Mylonis E. (2022). Effects of a futsal game on metabolic, hormonal, and muscle damage indicators of male futsal players. J. Strength Cond. Res..

[8] Floriano L.T., da Silva J.F., Teixeira A.S., Salvador P.C.d.N., Dittrich N., Carminatti L.J., Nascimento L.L., Guglielmo L.G.A. (2016). Physiological responses during the time limit at 100% of the peak velocity in the Carminatti’s test in futsal players. J. Hum. Kinet..

[9] Álvarez J.C.B., D’ottavio S., Vera J.G., Castagna C. (2009). Aerobic fitness in futsal players of different competitive level. J. Strength Cond. Res..

[10] Barcelos R.P., Tocchetto G.L., Lima F.D., Stefanello S.T., Rodrigues H.F.M., Sangoi M.B., Moresco R.N., Royes L.F.F., Soares F.A.A., Bresciani G. (2017). Functional and biochemical adaptations of elite level futsal players from Brazil along a training season. Medicina.

[11] De Freitas V.H., Pereira L.A., de Souza E.A., Leicht A.S., Bertollo M., Nakamura F.Y. (2015). Sensitivity of the Yo-Yo intermittent recovery test and cardiac autonomic responses to training in futsal players. Int. J. Sports Physiol. Perform..

[12] Nakamura F.Y., Antunes P., Nunes C., Costa J.A., Esco M.R., Travassos B. (2020). Heart rate variability changes from traditional vs. ultra-short-term recordings in relation to preseason training load and performance in futsal players. J. Strength Cond. Res..

[13] Nakamura F.Y., Pereira L.A., Cal Abad C.C., Kobal R., Kitamura K., Roschel H., Rabelo F., Souza W.A., Loturco I. (2015). Differences in physical performance between U-20 and senior top-level Brazilian futsal players. J. Sports Med. Phys. Fit..

[14] De Freitas V.H., Rinaldo M., Turquino G.G., Miloski B., Ramos S.d.P. (2019). Training aimed at the development of power and physical performance of futsal players. Rev. Bras. Cineantropometria Desempenho Hum..

[15] Galy O., Zongo P., Chamari K., Michalak E., Dellal A., Castagna C., Hue O. (2014). Anthropometric and physiological characteristics of Melanesian futsal players: A first approach to talent identification in Oceania. Biol. Sport.

[16] De Moura N.R., Borges L.S., Santos V.C., Joel G.B., Bortolon J.R., Hirabara S.M., Cury-Boaventura M.F., Pithon-Curi T.C., Curi R., Hatanaka E. (2013). Muscle lesions and inflammation in futsal players according to their tactical positions. J. Strength Cond. Res..

[17] Jovanovic M., Sporis G., Milanovic Z. (2011). Differences in situational and morphological parameters between male soccer and futsal—A comparative study. Int. J. Perform. Anal. Sport.

[18] Garrido-Chamorro R., Sirvent-Belando J.E., González-Lorenzo M., Blasco-Lafarga C., Roche E. (2012). Skinfold sum: Reference values for top athletes. Int. J. Morphol..

[19] Naser N., Ali A. (2016). A descriptive-comparative study of performance characteristics in futsal players of different levels. J. Sports Sci..

[20] López-Fernández J., García-Unanue J., Sánchez-Sánchez J., Colino E., Hernando E., Gallardo L. (2020). Bilateral asymmetries assessment in elite and sub-elite male futsal players. Int. J. Environ. Res. Public Health.

[21] Pedro R.E., Milanez V.F., Boullosa D.A., Nakamura F.Y. (2013). Running speeds at ventilatory threshold and maximal oxygen consumption discriminate futsal competitive level. J. Strength Cond. Res..

[22] Nughes E., Rago V., Aquino R., Ermidis G., Randers M.B., Ardigò L.P. (2020). Anthropometric and functional profile of selected vs. non-selected 13-to-17-year-old soccer players. Sports.

[23] Aquino R., Alves I.S., Padilha M.B., Casanova F., Puggina E.F., Maia J. (2017). Multivariate profiles of selected versus non-selected elite youth Brazilian soccer players. J. Hum. Kinet..

[24] Miloski B., Moreira A., Andrade F.C., Freitas V.H., Peçanha T., Nogueira R.A., Bara-Filho M. (2014). Do physical fitness measures influence internal training load responses in high-level futsal players?. J. Sport. Med. Phys. Fit..

[25] Milanez V.F., Pedro R.E., Moreira A., Boullosa D.A., Salle-Neto F., Nakamura F.Y. (2011). The role of aerobic fitness on session rating of perceived exertion in futsal players. Int. J. Sports Physiol. Perform..

[26] Faulkner J.A., Falls H.B. (1968). Physiology of swimming and diving. Exercise Physiology.

[27] Hockey R.V. (1993). Physical Fitness: The Pathway to Healthful Living.

[28] Hickox L.J., Ashby B.M., Alderink G.J. (2016). Exploration of the validity of the two-dimensional sagittal plane assumption in modeling the standing long jump. J. Biomech..

[29] Krustrup P., Mohr M., Amstrup T., Rysgaard T., Johansen J., Steensberg A., Pedersen P.K., Bangsbo J. (2003). The Yo-Yo intermittent recovery test: Physiological response, reliability, and validity. Med. Sci. Sports Exerc..

[30] Markovic G., Dizdar D., Jukic I., Cardinale M. (2004). Reliability and factorial validity of squat and countermovement jump tests. J. Strength Cond. Res..

[31] Silva R., Rico-González M., Lima R., Akyildiz Z., Pino-Ortega J., Clemente F.M. (2021). Validity and reliability of mobile applications for assessing strength, power, velocity, and change-of-direction: A systematic review. Sensors.

[32] Buchheit M. (2008). The 30-15 intermittent fitness test: Accuracy for individualizing interval training of young intermittent sport players. J. Strength Cond. Res..

[33] Borg G. (1998). Perceived Exertion and Pain Scales.

[34] Foster C., Florhaug J.A., Franklin J., Gottschall L., Hrovatin L.A., Parker S., Doleshal P., Dodge C., Hrovation L., Parker S. (2001). A new approach to monitoring exercise training. J. Strength Cond. Res..

[35] Akyildiz Z., Yildiz M., Clemente F.M. (2020). The reliability and accuracy of Polar Team Pro GPS units. Proc. Inst. Mech. Eng. Part P J. Sports Eng. Technol..

[36] Ramos-Campo D.J., Rubio-Arias J.A., Carrasco-Poyatos M., Alcaraz P.E. (2016). Physical performance of elite and subelite Spanish female futsal players. Biol. Sport.

[37] Ferraz R., Gonçalves B., Coutinho D., Oliveira R., Travassos B., Sampaio J., Marques M.C. (2020). Effects of knowing the task’s duration on soccer players’ positioning and pacing behaviour during small-sided games. Int. J. Environ. Res. Public Health.

[38] Renfree A., Martin L., Micklewright D., St Clair Gibson A. (2014). Application of decision-making theory to the regulation of muscular work rate during self-paced competitive endurance activity. Sport. Med..

[39] Ferraz R., Gonçalves B., Coutinho D., Marinho D.A., Sampaio J., Marques M.C. (2018). Pacing behaviour of players in team sports: Influence of match status manipulation and task duration knowledge. PLoS ONE.

[40] Berdejo del Fresno D. (2012). Fitness seasonal changes in a first division English futsal. Afr. J. Basic Appl. Sci..

